# Physical fitness and quality of life in Austrian adolescents post-COVID-19: A one-year longitudinal study

**DOI:** 10.3934/publichealth.2026048

**Published:** 2026-08-27

**Authors:** Vera Prünster, Martin Niedermeier, Armando Cocca, Gerhard Ruedl

**Affiliations:** 1 Department of Sport Science, University of Innsbruck, 6020 Innsbruck, Austria; 2 Department of Human Movement Sciences, University of Ostrava, Czech Republic

**Keywords:** physical fitness, health-related quality of life, physical well-being, psychological well-being, autonomy and parent relationship, peers and social support, school environment

## Abstract

**Aims:**

Health-related quality of life (HRQoL) reflects adolescents' overall well-being across physical, psychological, social, and emotional dimensions. Pre-pandemic studies show positive associations between physical fitness (PF) and HRQoL, but the COVID-19 pandemic significantly reduced adolescents' physical activity, PF, and mental well-being. Although adolescents are consistently identified as a highly affected group, longitudinal research on the relationship between (the development of) PF and HRQoL during mid-adolescence remains scarce. This study aimed to investigate the prospective relationship between (changes in) PF and HRQoL over 12 months in upper secondary school adolescents following the easing of COVID-19 restrictions.

**Methods:**

A total of 140 adolescents (54.9% female; mean age 14.3 ± 0.5 years) participated in the present longitudinal study with two data collection time points during (October 2021) and after (October 2022) the pandemic restrictions. The participants completed the KIDSCREEN-10 (KS10) questionnaire and the KIDSCREEN-27 (KS27) questionnaire — comprising physical well-being, psychological well-being, autonomy and parent relations, social support and peers, and school environment — and underwent the standardized German Motor Performance Test (DMT 6–18), including eight tests to assess endurance, strength, speed, flexibility, and coordination.

**Results:**

Adolescents' HRQoL did not change significantly across the study period; PF significantly improved in six out of eight DMT test items over time. In multiple regression analyses, improvements in PF test items over the 12-month period were not significantly associated with overall HRQoL (KS10) after the restrictions, whereas HRQoL during the restrictions and male sex emerged as significantly negative associated variables, with male sex being significantly associated with the overall HRQoL index (KS10) but not with any of the five specific HRQoL dimensions (KS27).

**Conclusion:**

These findings show that adolescents' HRQoL did not change significantly over the post-pandemic follow-up period, despite notable improvements in PF. These improvements alone may not be sufficient to enhance HRQoL in the aftermath of the pandemic. Instead, adolescents' baseline well-being and demographic factors such as sex appear to play a decisive role.

## 1. Introduction

Health-related quality of life (HRQoL) among children and adolescents is a central concept in pediatric and adolescent health research [1]. In addition to physical well-being, it encompasses psychological, social, and emotional aspects of daily life, thereby reflecting the subjective perception of one's health status [1]. In light of increasing societal, educational, and familial demands, the assessment and promotion of HRQoL are gaining importance, not least because HRQoL serves as a key indicator of overall well-being [2]. A differentiated analysis of HRQoL during adolescence enhances our understanding of current stressors and provides valuable insights for preventive and health-promoting interventions.

The relevance of HRQoL became particularly evident during the COVID-19 pandemic, which impacted the lives of young people in multifaceted ways [2]. School closures, social isolation, the cancellation of leisure activities, and family-related stressors contributed to a decline in mental well-being and an increase in symptoms of anxiety, depression, and stress among adolescents [2],[3]. For example, a German study reported that at least 30% of adolescents experienced psychological problems such as anxiety and depression symptoms during the first lockdown phase, compared to 18% before the pandemic [4]. Research indicates that psychosocial dimensions of HRQoL — such as perceived social support, emotional experiences, and self-efficacy — were significantly impaired during the pandemic [2]. Adolescents from socioeconomically disadvantaged backgrounds or with preexisting mental health problems were particularly vulnerable [2],[5],[6]. These developments underscore the necessity of long-term preventive strategies to stabilize and promote HRQoL beyond acute crises.

In addition to psychological and social factors, physical fitness (PF) is increasingly recognized as a key determinant of adolescents' health and well-being [7]. According to a systematic review and meta-analysis by Bermejo-Cantarero et al. [8], both cardiorespiratory fitness and muscular fitness are positively associated with HRQoL, particularly in the domains of physical well-being, psychological well-being, and peer relations. Furthermore, their results indicate that cardiorespiratory fitness is positively linked to the school environment, while muscular fitness shows a positive association with family relations [8]. High levels of cardiorespiratory endurance, muscular strength, and coordination are closely associated with a healthy cardiovascular system, lower risk of overweight, and improved motor development [9]. Furthermore, numerous studies suggest that physically fit adolescents are less likely to experience psychological distress and tend to exhibit higher levels of self-esteem [7],[10],[11]. PF also plays an essential role in academic contexts, as it has been linked to enhanced cognitive abilities, such as attention, working memory, and executive functioning [12],[13]. Moreover, sports activities foster social skills such as teamwork, empathy, and communication, thereby contributing to psychosocial integration [14],[15]. Overall, PF is a fundamental pillar of holistic healthy development during adolescence [16]. These effects are particularly important during puberty, when adolescents experience rapid neurodevelopmental changes affecting emotion regulation, motivation, and social behavior [17].

However, the COVID-19 pandemic not only affected adolescents' mental health but also had a substantial impact on their physical activity levels as well as on their PF [18]–[21]. The closure of schools, sports facilities, and sports clubs, especially during lockdown periods, significantly reduced regular physical activity and PF in children and adolescents [18],[19].

Numerous cross-sectional studies have demonstrated a strong association between PF and HRQoL in adolescents [22],[23]. In particular, cardiorespiratory endurance and muscular strength show positive correlations with various HRQoL dimensions such as physical and mental well-being, perceived social support, and school-related experiences [22],[23]. Comparative findings from studies in different countries, such as Norway, Germany, and Japan, indicate that this association is robust across diverse cultural contexts [5],[22],[24]. For instance, a study by Leibinger et al. [22] found that Norwegian adolescents with higher endurance and trunk strength scored higher on KIDSCREEN-27 dimensions. Psychological factors such as self-efficacy and life satisfaction have also been shown to be associated with an active lifestyle in adolescence [25],[26]. This can be theoretically embedded in behavioral models such as the Self-Determination Theory [27], which emphasizes the interaction between physical activity, intrinsic motivation, and psychological well-being [28] and posits that engagement in physical activity and improvements in PF may enhance the basic psychological need for competence, thereby contributing to higher HRQoL. These cross-sectional findings highlight the central role of physical activity and PF in the well-being of adolescents.

However, to examine causal relationships between PF and HRQoL, longitudinal studies are needed [22]. Longitudinal studies conducted before the onset of the COVID-19 pandemic consistently demonstrate positive associations between PF and HRQoL [29]–[33]. A longitudinal study by Evaristo et al. [29] indicates that a decline in cardiorespiratory fitness is associated with a deterioration in HRQoL, as assessed using the KIDSCREEN-10 questionnaire, over a two-year period in Portuguese adolescents aged 12–18 years. Solera-Sanchez et al. [32] investigated the combined influence of various PF components on HRQoL in approximately 200 Spanish adolescents with a mean age of 13.9 years. The findings indicated that adolescents with a higher PF index — comprising cardiorespiratory fitness, muscular strength, and motor competence — at baseline were more likely to exhibit greater HRQoL at follow-up [32]. The results of a 12-week longitudinal study involving English primary school children with a mean age of 10 years also demonstrated a positive association between cardiorespiratory fitness at baseline and HRQoL at the 12-week follow-up [33].

There is global evidence indicating that the restrictions imposed due to the COVID-19 pandemic have led, particularly among children and adolescents, not only to a reduction in HRQoL [34], physical activity [35],[36], and PF but also to an increase in sedentary behavior [35]–[37], overweight [37],[38], and mental health problems [39]. Therefore, longitudinal studies that capture pre- and post-pandemic data are essential to better understand how the COVID-19 restrictions have affected the relationship between PF and HRQoL among adolescents, and whether these effects persist, diminish, or intensify over time. However, to date, there are very few studies addressing this topic. Basterfield et al. [31] assessed HRQoL, PF, and sports club participation in a sample of 178 English primary school children aged 8–10 years, both before and after the first COVID-19 lockdown in 2020. The authors observed no significant changes in the mean HRQoL domains from baseline to follow-up [31]. Accordingly, at the group level, there were no associations between changes in HRQoL and changes in PF variables [31]. However, at the individual level, children with the greatest increase in weight status reported the largest decline in the physical well-being domain [31]. In a follow-up publication, Basterfield et al. [30] published the results of a total of 90 children (aged 8–9 years) assessed at four time points before, during, and after the COVID-19 pandemic: October 2019, November 2020, November 2021, and June 2022. The study found no association between PF and HRQoL, but physical well-being decreased over time, while autonomy and parent relations increased [30]. In addition, psychological well-being and social support and peers showed no significant changes, and the school environment remained stable except for a decline among girls [30]. Regarding PF, endurance first declined and then improved beyond baseline, strength increased continuously, and flexibility dropped after the lockdown but later returned to baseline [30].

Such studies are rare for mid-adolescence (14–17 years), but numerous studies have shown that mid-adolescents are the age group most affected by the COVID-19 pandemic and its consequences [40]–[43]. COVID-19 had strong negative effects on adolescents' mental health and well-being worldwide [40]–[43]. Samji et al. [41] reported in their systematic review that older adolescents suffered more from depression, stress, anxiety, and worry than younger children, while younger children showed higher rates of hyperactivity and behavioral problems. During this period, puberty-related hormonal changes may influence physical fitness development and responsiveness to environmental or contextual factors [44]. At the same time, adolescents face increasing academic demands, which can reduce opportunities for physical activity and alter lifestyle patterns [45]. Moreover, peer relationships and the social context of sports gain heightened importance, potentially shaping motivation, engagement, and responses to changes in physical fitness [46]. These studies also emphasize the need for robust longitudinal research.

To the best of our knowledge, there is limited research examining the longitudinal associations between HRQoL and PF in mid-adolescence following the lifting of COVID-19 pandemic restrictions in Austria. Therefore, the present study aimed to analyze the prospective relationships between (changes in) PF development and the various dimensions of HRQoL (physical well-being, psychological well-being, autonomy and parent relations, social support and peers, and school environment) development over 12 months in Austrian upper secondary school adolescents.

## 2. Materials and methods

### 2.1. Study design and participants

The data collection of this longitudinal study took place between October 2021 and December 2022 at four secondary schools (upper level) in Tyrol, Austria. These schools were selected for participation using convenience sampling, based on existing administrative contacts. Inclusion criteria required students to be enrolled in the 9th grade at the time of the initial assessment and to provide informed consent. Schools were selected using convenience sampling based on existing administrative contacts and geographic proximity to Innsbruck, which enabled standardized data collection by trained university staff. To enhance comparability, only schools with similar curricular structures (i.e., comparable weekly instructional time) were included. Within participating schools, all students enrolled in the 9th grade were invited to participate; no additional exclusion criteria were applied, and participation was based solely on informed consent. This approach resulted in a regionally bounded but structurally comparable sample of Tyrolean adolescents.

The study consisted of two waves of data collection. The first wave was conducted between October and December 2021 (T1), while the second wave occurred between October and December 2022 (T2).

In total, 257 adolescents participated in the first assessment wave (T1). Of these, 140 adolescents (54.9% girls) provided informed consent and completed the full set of questionnaires and physical performance tests at both time points and were therefore included in the longitudinal dataset. A substantial proportion of this attrition can be explained by structural factors in the Austrian education system, as compulsory schooling ends after grade 9 (i.e., between T1 and T2), leading some students to leave the school context and become unavailable for follow-up; additional losses were likely due to temporary absences (e.g., illness) at the second assessment wave.

Written informed consent was obtained from the legal representatives and the assent of the adolescents. Before data collection, ethical approval was obtained from the Tyrolean Education Directorate, the Ethics Advisory Board of the University of Innsbruck (Certificate of Good Standing, 73/2021), and the principals of the participating schools.

### 2.2. Test procedures

The instruments and procedures used to assess overall HRQoL, various dimensions of HRQoL, and PF were consistent across both data collection waves to ensure comparability over time.

#### 2.2.1. Health-related quality of life and its dimensions

HRQoL and its dimensions were assessed using the KIDSCREEN-27 (KS-27) questionnaire, which was developed for children and adolescents aged 8 – 18 years [1]. It consists of 27 items, each rated on a 5-point Likert scale ranging from 1 (“not at all”) to 5 (“extremely”) [1]. The KS-27 measures five dimensions of HRQoL: physical well-being (five items), psychological well-being (seven items), autonomy and parent relations (seven items), peers and social support (four items), and school environment (four items) [1].

Ten of these twenty-seven items comprise the KIDSCREEN-10 (KS-10) index, which was used to measure overall HRQoL [1]. After recoding negatively scored items, raw scores were summed and converted into standardized T-scores using RASCH-based person parameter estimates [1], with higher T-scores reflecting greater HRQoL. Higher T-scores indicate a better perceived HRQoL [1]. Its reliability and validity have been extensively tested and confirmed [1],[47]. Cronbach's alpha is 0.82 for overall HRQoL (KS-10) and ranges from 0.79 (physical well-being) to 0.84 (psychological well-being) for the five dimensions of the KS-27 [47]. In the present study, Cronbach's alpha values were 0.62 (T1) and 0.70 (T2) for overall HRQoL, 0.55 (T1) and 0.56 (T2) for physical well-being, 0.84 (T1) and 0.85 (T2) for psychological well-being, 0.78 (T1) and 0.87 (T2) for autonomy and parent relations, 0.78 (T1) and 0.83 (T2) for peers and social support, and 0.82 (T1) and 0.86 (T2) for school environment. However, reliability for the physical well-being subscale was relatively low in the present sample, which should be considered when interpreting results.

Participants whose T-scores exceeded the European normative averages — 53.90 in overall HRQoL (KS-10 index) as well as 53.72 in physical well-being, 53.04 in psychological well-being, 51.57 in autonomy and parents, 51.00 in social support and peers, and 54.03 in school environment — were classified as having high HRQoL [1]. Scores above these cutoff values are associated with a positive psychological profile, including feelings of happiness, life satisfaction, optimism, and emotional stability [1]. Conversely, lower T-scores are linked to reduced well-being, characterized by symptoms such as sadness, low self-esteem, and a general lack of enjoyment in life [1].

#### 2.2.2. Physical fitness

PF was measured using the German Motor Test (DMT) for children and adolescents aged 6 – 18 years [48]. This standardized test battery comprises eight subtests assessing different components of PF [48]: a 20-m sprint (speed), backward balancing on three beams of varying widths (coordination requiring precision), jumping sideways over a center line for 15 s (coordination under time constraints), a trunk bend forward test (flexibility), push-ups performed over 40 s (muscular endurance), sit-ups over 40 s (muscular endurance), a standing long jump (explosive strength), and a 6-min run (aerobic endurance). As reported by Bös et al. [48], the battery demonstrates strong inter-rater reliability (*r* = 0.95) and good test–retest reliability (*r* = 0.82), and it is validated for measuring speed, coordination, flexibility, strength, and endurance. The raw scores of the test items from T1 were standardized, and the test items from T2 were standardized using the means from T1. Subsequently, a difference variable was calculated for each item by subtracting T1 from T2. Given the heterogeneity in the development of the subtests, no composite score for physical fitness was used.

### 2.3. Statistics

Statistical analyses were performed using IBM SPSS version 29.0.0.0. Descriptive statistics (means ± standard deviations, medians and interquartile ranges, and absolute and relative frequencies) were calculated for all variables. Sex was coded with females as the reference category. Normality was assessed using the Shapiro–Wilk test. Depending on the distribution of the data, paired t-tests or Wilcoxon signed-rank tests were used to evaluate within-subject changes between the two measurement points for KS-10 and KS-27, including five dimensions — physical well-being, psychological well-being, autonomy and parent relations, social support and peers, and school environment — as well as the raw scores of the PF tests. Effect sizes as Cohen's d with the conventions small (0.2), medium (0.5), and large (0.8) or Rosenthal's effect size r with the conventions small (0.1), medium (0.3), and large (0.5) were used as appropriate [49].

Subsequently, a series of multiple linear regression analyses with HRQoL and the dimensions of HRQoL of T2 were conducted to analyze the development of the PF subtests, taking into account relevant covariates. Six separate multiple linear regression models were calculated with HRQoL and the dimensions of HRQoL of T2 as dependent variables. HRQoL and the respective dimension of HRQoL at T1 were included in the model as a covariate. Furthermore, based on previous research, regression models were adjusted for the covariate sex [50]. The explained variance was interpreted based on Cohen's benchmarks: *R^2^* = 0.02 (small), *R^2^* = 0.13 (moderate), and *R^2^* = 0.26 (large) [49]. A *p*-value < 0.050 was considered statistically significant in all tests (two-tailed). Given the exploratory nature of the regression analyses across multiple HRQoL dimensions, no correction for multiple comparisons was applied.

## 3. Results

### 3.1. Sample characteristics and development of HRQoL and PF

Among the 140 adolescents in the present school sample, the average age was 14.3 ± 0.5 years (T1), and the average body mass index was 20.52 ± 3.24 kg/m^2^ (T1) and 21.24 ± 3.04 kg/m^2^ (T2). In total, 53.6% of the participants were female.

Attrition analyses revealed no significant baseline differences in PF between completers and non-completers. However, non-completers reported significantly lower baseline HRQoL scores in overall HRQoL (KS-10 index), physical well-being (KS-27), psychological well-being (KS-27), parent relations (KS-27), and school environment (KS-27), although effect sizes were small to moderate (*d* = 0.28–0.43).

Table 1 compares the mean values of overall HRQoL (KS-10 index) and all five dimensions of HRQoL (KS-27) of T1 and T2. No significant differences were found between T1 and T2 in overall HRQoL and all dimensions of HRQoL.

**Table 1.** Comparison of overall HRQoL (KS-10 index) and five dimensions of HRQoL (KS-27) between T1 and T2.

	*Mean* ± *SD* T1	*Mean* ± *SD* T2	*Median* T1	IQR T1	*Median* T2	IQR T2	*p*-value	Effect size
HRQoL	49.64 ± 8.73	49.60 ± 10.00	49.76	9.76	49.76	11.72	0.708^a^	*d* = 0.06 *r* = 0.03
Phy-WB	48.67 ± 9.32	48.09 ± 9.71	47.08	13.07	47.08	13.07	0.574^b^	*d* = 0.05
Psy-WB	48.15 ± 9.13	48.15 ± 10.16	48.45	11.32	48.45	12.69	0.426^b^	*d* = −0.09
Parents	54.93 ± 9.64	55.07 ± 11.93	53.25	11.83	53.25	16.06	0.998^b^	*d* < 0.01
Peers	50.80 ± 8.86	51.67 ± 10.01	53.23	10.91	53.23	10.91	0.131^a^	*d* = 0.26 *r* = 0.13
School	51.66 ± 9.60	49.83 ± 10.31	51.08	12.78	51.08	13.34	0.300^b^	*d* = 0.10

Note: *p < 0.05; IQR = interquartile range; HRQoL = health-related quality of life (overall index); Phy-WB = physical well-being; Psy-WB = psychological well-being; Parents = autonomy and parent relations; Peers = social support and peers; School = School environment; ^a^ Wilcoxon test; ^b^ *t*-test.

Table 2 compares the items of the German Motor Test (DMT 6-18) at T1 and T2. No significant difference was observed in the 6-min run. Sprint performance significantly decreased (indicated by a significantly increased sprint time), while the tests balancing backward, jumping, trunk flexion, push-ups, sit-ups, and standing long jump showed significant improvements from T1 to T2.

### 3.2. Associations between HRQoL and the development of PF

Multicollinearity diagnostics revealed acceptable variance inflation factor (VIF) values for all predictors (all VIFs < 2.5), indicating no evidence of problematic multicollinearity.

Table 3 summarizes the results of the multiple linear regression analyses with overall HRQoL (KS-10) and its dimensions at T2 as dependent variables. Overall HRQoL/the respective dimension of HRQoL at T1 are significant independent predictors of HRQoL/the respective dimension of HRQoL at T2, with p < 0.001. Changes in the PF item (Δ *z*-score) *6-min run* showed significant positive associations with physical well-being (KS-27) at T2. Improvements in sit-ups and decreases in standing long jump performance were significantly associated with higher values in the school environment dimension across 12 months. Male sex was significantly associated with higher overall HRQoL and higher values in the dimension of school environment.

**Table 2.** Comparison of physical fitness (absolute values) between T1 and T2.

	*Mean* ± *SD* T1	*Mean* ± *SD* T2	*Median* T1	IQR T1	*Median* T2	IQR T2	p-value	Effect size
Sprint (s)	3.63 ± 0.38	3.80 ± 0.38	3.65	0.56	3.80	0.56	<0.001*^b^	d = 0.55
Balancing backward (steps)	35.58 ± 11.86	41.33 ± 7.44	37.00	19.00	44.00	10.00	<0.001*^b^	d = 0.56
Jumping (reps)	39.63 ± 12.58	50.65 ± 6.18	43.00	10.5	50.50	8.0	<0.001*^b^	d = 0.95
Trunk flexion (cm)	4.03 ± 10.23	5.59 ± 9.86	6.00	14.2	6.50	11.4	0.034*^b^	d = 0.18
Push-ups (count)	16.13 ± 8.00	17.66 ± 3.32	16.00	6.0	18.00	5	<0.001*^a^	d = 0.70 r = 0.33
Sit-ups (count)	23.89 ± 8.01	27.04 ± 6.01	24.00	7.0	26.00	7	<0.001*^a^	d = 0.93 r = 0.42
St. long jump (cm)	171.11 ± 49.38	185.33 ± 34.30	175.00	50.0	183.00	52	<0.001*^a^	d = 0.68 r = 0.32
6-min run (m)	1045.55 ± 378.59	1083.79 ± 174.45	1081.00	301.0	1076.00	236	0.532^a^	d = 0.10 r = 0.05

Note: *p < 0.05; IQR = interquartile range; Jumping = jumping sideways; St. long jump = standing long jump; ^a^ Wilcoxon test; ^b^ *t*-test.

**Table 3.** Results of the multiple linear regression analyses on the dependent variables health-related quality of life and its dimensions (physical well-being, psychological well-being, autonomy and parent relations, peers and social support, and school environment).

Dependent variable	HRQoL T2		Phy-WB T2		Psy-WB T2		Parent T2		Peers T2		School T2	

	*β*	p-value	*β*	p-value	*β*	p-value	*β*	p-value	*β*	p-value	*β*	p-value
Intercept		<0.001*		<0.001*		<0.001*		0.001*		<0.001*		<0.001*
HRQoL/respective dimension of HRQoL (T1)	0.51	<0.001*	0.47	<0.001*	0.44	<0.001*	0.52	<0.001*	0.44	<0.001*	0.46	<0.001*
Δ *z*-score sprint	−0.05	0.578	−0.04	0.664	−0.10	0.273	−0.02	0.789	0.42	0.623	−0.03	0.726
Δ *z*-score bal. bw.	0.08	0.393	0.02	0.830	0.11	0.241	0.08	0.344	−0.15	0.095	0.11	0.188
Δ *z*-score jump.sw.	−0.02	0.829	−0.03	0.822	−0.06	0.610	−0.05	0.617	0.19	0.080	0.02	0.886
Δ *z*-score tr.flex.	−0.02	0.775	0.13	0.124	−0.11	0.211	−0.06	0.480	−0.10	0.242	−0.07	0.356
Δ *z*-score push ups	−0.05	0.580	−0.03	0.714	−0.10	0.282	−0.07	0.441	0.05	0.586	−0.04	0.648
Δ *z*-score sit ups	0.08	0.505	0.10	0.379	0.14	0.250	<0.01	0.980	−0.09	0.411	0.23	0.029*
Δ *z*-score st.l.jump	−0.17	0.191	−0.10	0.433	−0.16	0.262	0.06	0.617	−0.03	0.832	−0.30	0.015*
Δ *z*-score 6min run	0.10	0.340	0.21	0.046*	0.13	0.223	−0.001	0.991	−0.03	0.810	0.01	0.922
Sex	0.22	0.014*	0.04	0.683	0.13	0.179	0.09	0.295	0.04	0.703	0.23	0.009*
*R^2^*	0.334		0.321		0.287		0.309		0.240		0.320

Note: *p < 0.05; HRQoL = health-related quality of life (overall index); Phy-WB = physical well-being; Psy-WB = psychological well-being; Parents = Autonomy and parent relations; Peers = social support and peers; School = school environment; bal.bw. = balancing backward; jump.sw. = jumping sideways; tr.flex. = trunk flexion; st.l.jump = standing long jump; beta = standardized regression coefficient; sex was coded with females as the reference category.

## 4. Discussion

In the present one-year study period, adolescents' overall HRQoL (KS-10 index), as well as the five dimensions of KS-27, did not significantly differ between the two measurement points. Regarding PF, while no changes were found in the endurance test, participants significantly improved in other performance tests except the sprint, where performance significantly declined. In the multiple regression analyses, the strongest predictor of HRQoL after the pandemic restrictions was HRQoL during the pandemic restrictions, both in overall HRQoL (KS-10 index) and in the five dimensions of the KS-27, while changes in PF did not significantly predict overall HRQoL at T2. Improvements in the 6-min run were significantly associated with higher physical well-being, and improvements in sit-up performance and decreases in standing long jump performance were significantly associated with the school environment dimension. In addition, male adolescents reported higher overall HRQoL values after the pandemic restrictions. Notably, substantial improvements in objective PF were not accompanied by corresponding improvements in self-reported physical well-being, which remained stable or slightly declined over time, highlighting a divergence between objective physical recovery and subjective well-being.

The present longitudinal study shows that the HRQoL of the adolescents surveyed remained largely stable over one year after the end of the COVID-19 restrictions in Austria. This result is consistent with previous findings suggesting that HRQoL values during adolescence are relatively stable over time and appear to return to this stability once major health or social disruptions (such as pandemic-related restrictions) have subsided [51]–[53]. In accordance, Basterfield et al. [30],[31] also observed that children's HRQoL remained mostly unchanged during and after the COVID-19 lockdowns in England, with the notable exception of decreased scores in physical well-being and increased scores in autonomy and parents. These findings underline that while certain domains of HRQoL appear robust even under the disruptive conditions of the pandemic, specific aspects such as physical well-being may still be negatively affected. In addition to Gillison et al. [53], Schmidt et al. [52] and Ellert et al. [51] also provide evidence that adolescents' HRQoL generally remains relatively stable over short periods of time (up to 1 year) and appears to return to this stability once major stressors, such as pandemic-related restrictions, have subsided. By contrast, most other studies with longer follow-up periods or during specific developmental phases (e.g., puberty) report declines in HRQoL [29],[32],[54],[55]. However, in relation to the European normative values, which define high HRQoL as exceeding these mean values, both overall HRQoL and the dimensions of physical well-being, psychological well-being, and school environment indicated low HRQoL levels both during and after the pandemic restrictions. This may be attributable to the restrictions imposed during the COVID-19 pandemic, such as school closures, social isolation from peers, and the cancellation of leisure activities. Conversely, adolescents clearly exceeded the normative values for the dimension “autonomy and parent relations” (51.57) both during (54.93) and after the pandemic restrictions (55.07). Furthermore, the normative values for the dimension “social support and peers” (51.00) were slightly below the normative value at T1 (50.80) and after the pandemic restrictions (51.67). A possible explanation for exceeding the normative values in the dimension *autonomy and parent relations* could be pandemic-related measures such as school closures and lockdowns, which led adolescents to spend significantly more time in the home environment, where the relationship with their parents may have been experienced as more supportive. This interpretation is supported by findings from Bülow et al. [56], who showed that adolescents spent significantly more time with their parents during the COVID-19 lockdown (from 8.6 to 12.7 h per day), while contact with friends was strongly reduced. Similarly, Ellis et al. [57] reported that physical isolation limited peer interactions and that online communication could only partially compensate, suggesting that peer relationships and their supportive functions became less influential during lockdown. Resilience factors, such as hope and sense of coherence, have been identified as key resources for maintaining HRQoL in times of crisis [58]. Tal-Saban and Zaguri-Vittenberg [58] demonstrated that resilience factors were identified as key predictors of HRQoL, independent of social or demographic factors, and emphasized that these resources played a protective and stabilizing role, particularly during the pandemic. However, stability is not necessarily positive: persistently low HRQoL in the domain of psychological well-being may be an early indicator of health vulnerability [55]. Possible explanations for why HRQoL did not increase after the lifting of restrictions include longer-term psychological aftereffects of the pandemic [59], educational backlogs [60], or the challenges of adapting to new and demanding situations once schools and social life resumed [61].

While adolescents' HRQoL remained largely stable across measurement points, changes in PF offer a complementary but differentiated perspective. Consistent with age-related developmental processes, participants showed improvements in most PF components over time, likely reflecting pubertal changes in body composition, neuromuscular coordination, and training-related factors [62]–[64]. However, not all PF domains followed this general trend. Mean endurance performance did not improve significantly, and sprint performance declined, deviating from the continuous age-related gains in cardiorespiratory fitness reported in previous research [62]. Pandemic-related restrictions, including school closures, reduced physical education, and limited access to organized sports may have disproportionately affected endurance- and high-intensity activities and thus attenuated expected group-level improvements.

Importantly, the absence of a mean increase in endurance performance does not preclude meaningful individual variability in change. Accordingly, multiple linear regression analyses indicated that, at the individual level, improvements in cardiorespiratory endurance (6-min run Δ*z*-score) were positively associated with physical well-being at follow-up, independent of baseline physical well-being and other covariates. This suggests that adolescents who were able to maintain or improve their aerobic capacity despite overall constrained conditions perceived benefits in their physical well-being.

Associations between changes in PF and the school environment dimension of HRQoL were heterogeneous. Improvements in *z*-scores of muscular endurance, assessed by sit-ups, were positively associated with perceived school environment, whereas gains in explosive lower-body strength, measured by the standing long jump, were negatively associated. Importantly, these opposing effects were confined to the school environment dimension and did not extend to overall HRQoL or other domains, suggesting context-specific rather than general effects. This interpretation is consistent with conceptual frameworks of HRQoL that describe the school environment as a domain capturing structurally and socially embedded experiences related to academic demands, performance evaluation, and teacher–student relationships rather than global well-being [1]. One possible explanation may be that different PF components interact with the school context through distinct psychosocial mechanisms. Muscular endurance may be associated with persistence, self-regulation, and sustained effort, which are positively valued within the school and physical education context and have been linked to greater perceived competence and autonomous motivation [65]. In contrast, improvements in highly performance-oriented and socially salient PF components such as explosive strength may be accompanied by increased social comparison and performance pressure, particularly in competitive physical education settings, which have been shown to negatively affect motivational and psychosocial outcomes in adolescents [66]. Moreover, visible physical performance attributes are closely tied to adolescents' physical self-concept and peer evaluation, potentially amplifying evaluative stress within the school context [67]. However, given the simultaneous inclusion of multiple PF variables in the regression models, the observed negative association should be interpreted with caution, as suppressor effects or shared variance between PF components may also have contributed to this finding, despite acceptable multicollinearity diagnostics (all VIF's < 2.5).

Overall, the present findings indicate that changes in PF over one year did not substantially influence subsequent overall HRQoL. Instead, baseline HRQoL emerged as the strongest and most consistent predictor of later HRQoL outcomes, supporting previous longitudinal evidence [31]. Basterfield et al. [31] similarly reported stable HRQoL trajectories and no significant associations between changes in PF and later HRQoL in primary school children. In contrast, other studies have shown that higher baseline levels of PF, particularly cardiorespiratory fitness and muscular strength, were associated with higher HRQoL at follow-up [32]. Together, these findings suggest that PF may be more relevant as a preexisting resource for future HRQoL than as a driver of short-term changes. However, the generally weak associations between PF components and the physical well-being dimension should be interpreted with caution, as the relatively low internal consistency of this subscale (Cronbach's alpha = 0.55–0.56) may have attenuated relationships and reduced the likelihood of detecting significant effects. Consequently, the lack of significant associations for most PF components may reflect either genuinely weak relationships or limitations in the scale's ability to capture physical well-being in the present sample reliably. This measurement limitation may partly explain why only the 6-min run emerged as a significant predictor of physical well-being. Furthermore, attrition analyses indicated that adolescents with lower baseline HRQoL were more likely to drop out between T1 and T2. Consequently, the final longitudinal sample may have been somewhat positively biased regarding psychological well-being, potentially limiting the generalizability of the findings and attenuating observed associations. Therefore, the observed stability of HRQoL over time should be interpreted with caution, as it may partly reflect a selective retention of adolescents with higher baseline well-being rather than true stability across the original cohort. These considerations are particularly important when interpreting findings obtained under the exceptional conditions following the COVID-19 pandemic.

Significant sex differences were observed only in overall HRQoL, with girls reporting lower scores. Michel et al. [68] also found significant sex differences; however, these were not limited to overall HRQoL but were also evident in specific dimensions such as psychological well-being, moods and emotions, self-perception, and physical well-being, with girls consistently reporting lower scores. According to Michel et al. [68], these sex-specific differences can be attributed to hormonal changes during puberty as well as social role expectations and coping mechanisms.

These findings also have practical implications for school-based health promotion and adolescent health interventions. Given that baseline HRQoL emerged as the strongest predictor of later well-being, early identification and support of adolescents with reduced HRQoL may be particularly important. Furthermore, the observed divergence between improvements in objective PF and stable subjective well-being suggests that physical fitness promotion alone may be insufficient to enhance adolescents' perceived HRQoL. Instead, school-based programs may benefit from integrating physical activity initiatives with psychosocial and mental health support strategies in order to promote both physical and emotional well-being. Previous evidence indicates that school-based programs integrating regular physical activity into the school day can produce small but significant improvements in PF — particularly cardiorespiratory fitness — and positive effects on HRQoL in adolescents [8],[52],[69]–[71]. While some interventions improved HRQoL even without marked increases in physical activity levels [70], higher cardiorespiratory and muscular fitness were consistently associated with better HRQoL, especially in physical and psychological well-being and social dimensions [8]. Overall, the present findings support the importance of multidimensional school-based interventions that combine physical activity promotion with broader psychosocial support to foster adolescent health and well-being.

Several limitations of this study should be noted, including the relatively small sample size, the use of a convenience sample, which limits the generalizability of the findings, the focus on only two measurement points, and the limited observation period of one year. In addition, attrition analyses indicated that adolescents with lower baseline HRQoL were more likely to discontinue participation between T1 and T2, suggesting potential selection bias. Consequently, the final longitudinal sample may not fully represent the original cohort, and the observed stability of HRQoL may therefore be overestimated. However, it should be noted that analyses on differences between completers and non-completers cannot fully exclude attrition bias, particularly if dropout was related to unobserved factors. Future research should investigate larger, more representative samples over more extended periods of time and incorporate qualitative approaches to better understand the subjective mechanisms underlying the statistical relationships between PF and HRQoL. The internal consistency values of individual subscales were lower than those reported in previous validation studies, particularly for the physical well-being dimension. Therefore, findings related to this subscale should be interpreted with caution, as reduced reliability may have attenuated associations and limited the detection of significant effects. In addition, it would be worthwhile to systematically test theoretical models such as Self-Determination Theory in order to more precisely identify the pathways through which PF influences HRQoL. Furthermore, there is an inherent risk of bias in self-report questionnaires — even when validated, as in the case of the KIDSCREEN-10 and KIDSCREEN-27 — due to factors such as reduced attention or inaccurate responses [72]. Despite these limitations, this study provides valuable initial insights, being the first to examine the relationships between PF development and dimensions of HRQoL in adolescents after the end of COVID-19 restrictions in Austria.

## 5. Conclusions

The results of this longitudinal study indicate that adolescents' HRQoL did not change significantly over the course of one year following the lifting of COVID-19 pandemic restrictions. Baseline HRQoL emerged as the strongest predictor of subsequent HRQoL across overall and all five dimensions. Although PF improved in several test items, these changes were largely unrelated to subsequent overall HRQoL, indicating that short-term PF gains alone may be insufficient to enhance perceived HRQoL in the post-pandemic context; rather, such improvements may need to be sustained over a longer period before translating into measurable changes in subjective well-being.

## Use of AI tools declaration

The authors declare they have not used Artificial Intelligence (AI) tools in the creation of this article.
